# One-Year Follow-Up of Weight Trajectories After the EDDY School-Based Nutrition and Physical Activity Program

**DOI:** 10.3390/children13040485

**Published:** 2026-03-31

**Authors:** Paula Moliterno, Kurt Widhalm

**Affiliations:** 1 Austrian Academic Institute for Clinical Nutrition, 1090 Vienna, Austria; moliterno@oeaie.org; 2 Division of Clinical Nutrition and Prevention, Department of Pediatrics, Medical University of Vienna, 1090 Vienna, Austria

**Keywords:** BMI, longitudinal follow-up, school-based intervention

## Abstract

**Highlights:**

**What are the main findings?**

- Children maintained stable SDS-BMI one year after the EDDY healthy intervention ended.
- Modest sex differences in waist-to-height trajectories were observed, with changes remaining within healthy ranges.

**What are the implications of the main findings?**

- School-based nutrition and physical activity programs can sustain healthy SDS-BMI post-intervention.
- Sex-specific analyses in future obesity prevention trials are needed, particularly during the transition to adolescence, to optimize long-term effectiveness.

**Abstract:**

**Background/Objectives**: The Effect of Diet and Exercise Training on Obesity and Secondary Disease Prevention and on the Lifestyle of Young Children (EDDY) study implemented a two-year school-based nutrition and physical activity program in a Viennese primary school to promote healthy weight trajectories. This follow-up examined whether anthropometric benefits persisted one year after program cessation without active intervention. **Methods**: Prospective longitudinal data from 36 intervention-group children (76.6% completion rate from baseline n = 47) were analyzed. Standardized measurements at baseline (T1), post-intervention (T2), and one-year post-intervention (T3) included BMI, SDS-BMI, and waist-to-height ratio by trained staff. Mixed-effects models evaluated changes over time with time, sex, baseline age, and baseline anthropometry as fixed effects. **Results**: At T3, median age was 10.5 years (IQR: 10.2–11.0). BMI increased consistently with normal growth (time effect *p* = 0.112), with a mean post-intervention increase of 0.70 kg/m^2^ (95% CI: 0.41–0.99; *p* < 0.001). SDS-BMI remained stable across periods (time effect *p* = 0.967; post-intervention change +0.013 SDS units, 95% CI: −0.178 to 0.109; *p* = 0.826). Waist-to-height ratio showed small changes (time effect *p* = 0.041) modified by baseline values, remaining within normal range. No sex-by-time interactions were observed (all *p* > 0.13). **Conclusions**: One year post-EDDY intervention, participating children maintained stable SDS-BMI and waist-to-height trajectories, reflecting sustained healthy growth. These results highlight the feasibility of maintaining healthy weight trajectories after the EDDY school-based nutrition and physical activity program and underscore the need for larger controlled trials to establish the long-term effectiveness of school-based programs.

## 1. Introduction

Childhood obesity continues to pose major public health challenges, with recent evidence highlighting the considerable long-term economic burden of early-onset obesity in Austria [1]. Data from COSI, the world’s largest childhood obesity surveillance initiative [2], revealed a non-significant increase in obesity prevalence among Austrian boys and a non-significant decrease among Austrian girls aged 7–9 years compared with the previous survey period [2]. A similar trend was observed in the EDDY study conducted in Vienna [3] underscoring the need for effective preventive strategies in childhood to alleviate future health and economic costs for both individuals and society [1].

To effectively tackle this complex problem, a call to urgently generate actions has been made in order to avoid the projection that by 2050, around one-third of the world’s children and adolescents live with overweight or obesity [4]. Overall, most preventive strategies have been implemented at the organizational level, particularly in schools [5]. Evidence suggests that interventions combining dietary education and increased physical activity can lead to short- or medium-term reductions in SDS-BMI compared with controls [5]. However, a recent review indicates that such benefits often diminish over time, with little or no difference at long-term follow-up (mean difference in SDS-BMI −0.02, 95% CI −0.06 to 0.01) [5]. The long-term effectiveness of lifestyle interventions in children is closely linked to behavioral mechanisms such as self-efficacy and motivation [6], which play a central role in initiating and sustaining changes in dietary habits and physical activity. When interventions are designed by setting realistic goals, translating theoretical knowledge into practice, and acknowledging even small achievements, children’s confidence can increase, positively influencing anthropometric outcomes and overall well-being [6]. Additionally, parental nutritional knowledge may also improve [7]. However, although school-based programs may foster these mechanisms, the absence of continued nutrition education, and additional physical activity once an intervention ends, can lead to unfavorable weight trajectories, as ongoing support and reinforcement diminish within an obesogenic environment that shapes health behaviors [8].

Developed between 2022 and 2024, the EDDY study (Effect of Diet and Exercise Training on Obesity and Secondary Disease Prevention and on the Lifestyle of Young Children) implemented a two-year interdisciplinary, school-based program in Vienna targeting obesity and related health outcomes in primary school children. Briefly, the EDDY project implemented four additional combined nutrition and physical activity education lessons per month, each lasting 1.5 h (in addition to the two weekly hours of physical activity education and the absence of formal nutritional education in their curricula) [9]. The intervention included scientific- and evidence-based content on diverse topics related to healthy eating and well-being, which aimed to promote sustainable improvements in dietary habits and to develop skills through age-appropriate, practical, hands-on activities such as cooking. Physical activity lessons were designed to support physical development through a playful program including motor coordination, balance, strength, full-body flexibility, and social interaction. During the course of the intervention, teachers were involved in planning to adapt content to specific needs, and parents participated through newsletters that reinforced the topic content developed during that period at home. Following the intervention, the effect on adiposity indicators was evaluated, showing that total body fat percentage exhibited a small, non-significant reduction (median −0.9%, IQR −2.3 to 1.9; *p* = 0.28), while BMI-SDS remained stable (median change −0.1, IQR −0.3 to 0.3; *p* = 0.62) [9]. In contrast, health-related quality of life, a construct that encompasses both physical and psychosocial well-being, improved significantly after the two-year intervention [9]. Previous reports have shown that children with overweight and obesity have a lower quality of life compared to those with normal weight [10]. On the other hand, when treatment is implemented, quality of life can improve even if BMI change is modest, and this improvement may empower children and raise their confidence to adopt and maintain healthier choices [11]. On this basis, it is hypothesized that improvements in quality of life may enhance motivation and self-efficacy for changing unhealthy behaviors, beyond weight-related outcomes, thereby contributing to the maintenance of behavior changes after the intervention has ended [12].

Although the short-term effectiveness of obesity prevention programs is well established, relatively few studies have investigated whether these benefits are sustained once active support ends [13]. Long-term follow-up is crucial to determine whether positive outcomes persist without ongoing intervention amid growth spurts and behavioral changing risks. Some studies have reported persistent BMI benefits after 2 [14,15] 2 ½ [16], 3 [17], and 8 [18] years of follow-up. However, a recent systematic review concluded that there is still no consistent evidence of long-term effects beyond one year in primary school-based obesity prevention programs [8]. Differences in sample size, attrition, intervention duration, and follow-up length likely contribute to the heterogeneity of findings [8]. Given the limited number of post-intervention follow-up studies and the absence of such data from Austria, the present study aims to assess the long-term (12-month post-intervention) effects of the two-year EDDY program on children’s weight trajectories. Understanding weight evolution after program cessation is critical to evaluating the durability of prevention efforts and to informing future program design. This study contributes new evidence on the maintenance of healthy weight trajectories and the need for continued strategies to achieve lasting benefits in pediatric populations, a school-based prevention approach.

## 2. Materials and Methods

### 2.1. Study Sample

This study employed a prospective longitudinal design with three measurement time points using the sample derived from the EDDY program (“Effect of sports and diet training to prevent obesity and secondary diseases and to influence young children’s lifestyle”), a school-based health promotion initiative launched from September 2022 to June 2024 with support from the Austrian Federal Ministry of Education, Science and Research. The program implemented a combined healthy nutrition and physical activity intervention comprising two additional hours of weekly education on nutrition or exercise. Detailed information about the study has been described in detail elsewhere [9]. The intervention school is located in a middle-socioeconomic-status neighborhood with above-average net income compared to Vienna overall, low unemployment rates, and moderate education levels featuring low university graduate shares (~19–25%) but strong vocational training [19]. Participants from one intervention school in Vienna were assessed at baseline (T1), immediately after completing the intervention (T2), and one year later (T3). The third assessment (T3) was conducted one year after the intervention ended, to evaluate the persistence of effects in the absence of continued intervention. Follow-up measurements (T3) were only performed in the intervention group due to financial restraints. Children were included in the study if parental consent to participate was given at each time point, if they were present during the measurements, and if they were in good physical health.

Participants with incomplete follow-up data (T3) were excluded from the longitudinal analysis. From n = 47 [8.1 (IQR: 7.7–8.4) years] children included at baseline, finally n = 36 (76.6%) children with complete outcome data were included. Reasons for exclusion included missing data, absence of parental consent to participate or being absent during the measurement day. Compared to those included, children excluded from the present analysis were older and showed higher frequency of overweight/obesity at the end of the intervention (T2) (Table S1). The selective attrition of older children and those with overweight/obesity at the end of the intervention may have introduced survivor-type bias [20], yielding a relatively healthier subsample at follow-up and potentially overestimating the stability of SDS-BMI at T3.

Approval for the study was granted by the Ethics Committee of Sigmund Freud University, Vienna (protocol code 1242-2025). Parents provided written informed consent for their child’s participation, which was entirely voluntary, as no incentives or compensation were offered.

### 2.2. Anthropometric and Body Composition Variables

Anthropometric and body composition measures were collected at baseline (T1) and immediately after completing the intervention (T2). However, measurements performed one year later after the end of the intervention (T3) included only weight, height and waist circumference due to logistical constraints. Children’s body weight was measured using a Tanita body composition electronic scale (MC-780MA, TANITA Corporation, Tokyo, Japan), and height was measured using a stadiometer (SECA 213, Hamburg, Germany), with the child standing without shoes [21]. Body mass index (BMI) (kg/m^2^) was calculated and transformed to age- and sex-specific percentiles to classify nutritional status [22]. Low weight was classified as <3rd percentile, normal weight ≥3rd percentile, and overweight ≥90th percentile. The obesity category was considered as the sum of the original categories of obesity (≥97th percentile) and extreme obesity (≥99.5th percentile) [22]. The low weight category was combined with the normal weight category. The BMI-SDS was calculated using the LMS method [22]. Waist circumference was measured using an inelastic tape (SECA 201, Hamburg, Germany) at the narrowest point between the last rib and the highest point of the iliac crest. The anthropometric index waist-to-height ratio (waist—cm/height—cm) was used to assess abdominal fat distribution. This measure was selected as an indicator of central adiposity because it adjusts waist circumference for height, thereby accounting for body size and growth. It has been shown to be a practical and age-independent marker of abdominal adiposity and to have good discriminatory ability for cardiometabolic risk factors associated with obesity in pediatric populations [23,24]. We additionally included the fat mass index [total fat mass (kg) to the square of height (m^2^)] as descriptive variable at baseline. Total body fat was obtained through body composition analysis performed at baseline (MC-780MA, TANITA Corporation, Tokyo, Japan). All measurements were performed by trained staff using standardized procedures.

### 2.3. Additional Descriptive Variables

Socioeconomic variables such as age and sex were assessed through self-administered questionnaires. With parental self-report on weight (kg) and height (cm) we calculated parental BMI (kg/m^2^) and further classified it as a binary variable (normal weight; overweight/obesity). Given the self-reported nature of these measures, reporting bias and potential underestimation of BMI cannot be excluded. Additionally, parents were asked to report their nationality. Regularity of breakfast and eating window times were assessed by self-report in questionnaires at baseline. The frequency of having breakfast was assessed during weekdays and weekends and further categorized as having “regular” breakfast intake when the participant declared to always have breakfast at home on all weekdays and weekends. If breakfast was skipped some days (or permanently) during the week and weekend; “irregular” breakfast intake was categorized [21]. The eating window was calculated based on the hours between each child’s first and last meal [21].

Variables such as parental self-reported weight and height, frequency of breakfast consumption, and eating window were assessed at baseline only for descriptive purposes and were not collected at T3.

### 2.4. Statistical Analysis

Descriptive analyses were performed by calculating medians, interquartile range (IQR), and the frequency and percentage distribution to summarize the participants’ anthropometric outcome variables by sex and according to time-point.

Differences between children by sex were assessed using Chi-square or Fisher’s exact tests for categorical variables and the Mann–Whitney U nonparametric test for continuous variables. Sex differences in anthropometric changes across study periods were examined using the Mann–Whitney U test. Effect sizes were calculated as rank-biserial correlations (*r*_rb_ = Z/√N), where Z is the standardized Wilcoxon statistic and N is the total number of observations with complete data. Effect sizes were interpreted as small, medium, or large according to Cohen’s guidelines for correlations (0.10 = small, 0.30 = medium, and 0.50 = large) [25].

Longitudinal changes in BMI, SDS-BMI, and abdominal adiposity (assessed by waist-to-height ratio) during the intervention and one-year after the intervention ceased were analyzed using mixed models for repeated measures. Fixed effects included time, sex, baseline age, and baseline anthropometric measurements, along with time-by-sex and time-by-baseline anthropometric interactions to test effect modification, and participants as random effects. The time-by-baseline-age interaction was tested and removed due to non-significance (*p* > 0.27; threshold *p* < 0.050) and low age variability (IQR = 7.58–8.35 years); this simplified the model without loss of fit.

Data analysis was conducted using SAS (https://www.sas.com/en_us/software/on-demand-for-academics.html, accessed on 1 March 2025) OnDemand for Academics (Cary, NC, USA), and a two-sided *p* < 0.050 was assigned as significant.

## 3. Results

The median age of all participants was 7.91 (IQR: 7.58–8.35) years at baseline, 9.56 (IQR: 9.23–10.00) years after completing the intervention (T2) and 10.51 (IQR: 10.18–10.95) years one year after the intervention ended (T3). At the beginning of the intervention, no differences were observed in anthropometric measurements and body fat index between boys and girls. Regularity of breakfast and eating window times were similar between sexes. Parental median reported BMI of mothers was classified as normal weight, while that of fathers as overweight, with no differences between boys and girls (Table 1).

**Figure 1:**
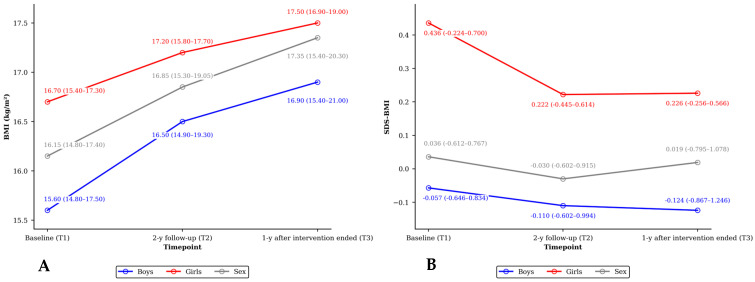
(**A**) Course of median (IQR) BMI over study periods by sex. (**B**) Course of median (IQR) SDS-BMI over study periods by sex.

BMI increased over time in growing children. Overall, median (IQR) BMI changed from 16.15 (14.80–17.40) kg/m^2^ at baseline (T1) to 16.85 (15.30–19.05) kg/m^2^ after the intervention (T2). One year after the intervention ended (T3), overall median (IQR) BMI was 17.35 (15.40–20.30) kg/m^2^. Median (IQR) SDS-BMI changed from 0.036 (–0.612–0.767) to −0.030 (–0.602–0.915) after the intervention, while after 1 year it was 0.019 (–0.795–1.078) (Figure 1).

At the end of the intervention period, 28 (77.8%) of the children were classified as having normal weight; 7 (19.4%) as overweight and 1 (2.8%) with obesity (differences between sexes *p* = 0.606). During the post-intervention period (T3–T2), 28 (77.8%) of the children remained in the normal weight category; 6 (16.7%) were classified as overweight and 2 (5.6%) with obesity. No differences were observed between sexes *p* = 0.910).

**Figure 2:**
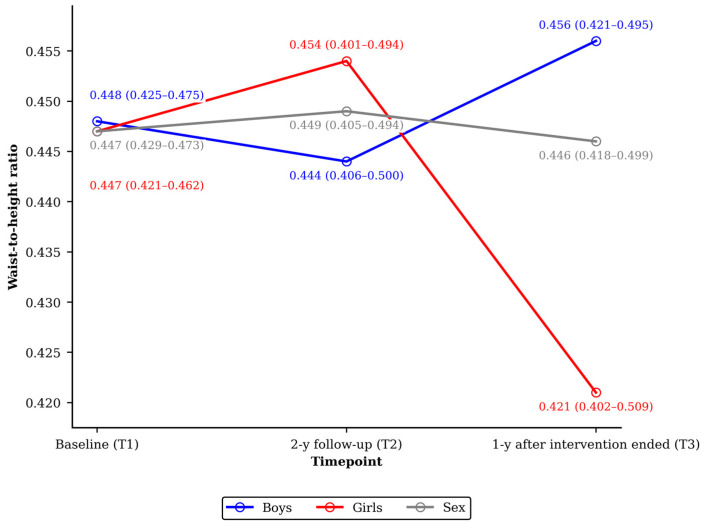
Course of median (IQR) waist-to-height ratio over study periods by sex.

Waist-to-height ratio was 0.447 (0.423–0.465); 0.449 (0.405–0.494) and 0.446 (0.418–0.499) in T1, T2 and T3, respectively. Waist circumference was 56.50 (55.00–61.50) cm at T1, 62.00 (57.50–68.00) cm at T2, and 64.00 (60.00–70.50) cm in T3. In girls and boys, respectively, it was 55.00 (54.00–63.00) cm and 57.00 (55.00–61.00) cm in T1, 61.00 (55.00–68.00) and 62.00 (58.50–68.00) cm in T2, and 61.00 (58.50–72.00) and 64.00 (60.00–70.00) cm at T3. Changes in waist circumference through the different study periods showed no statistical differences between sexes (T2–T1 *p* = 0.656; T3–T2 *p* = 0.882 and T3–T1 *p* = 0.113). Median BMI, SDS-BMI and waist-to-height ratio changes by sex across study periods are shown in Table 2. Nonparametric analyses showed small and non-significant sex differences across change periods (*r*_rb_ < 0.100, *p* ≥ 0.564; Table 2). An exception was observed for waist-to-height ratio change over the entire study period, which showed a small sex effect (*r*_rb_ = −0.324) at the threshold of statistical significance (*p* = 0.050), suggesting a modest tendency toward a greater reduction in girls compared with boys (Table 2; Figure 2).

Mixed models showed no evidence of overall significant changes in BMI over the study periods (time effect: F = 2.26, *p* = 0.112, Table 3). However, a significant modification of BMI trajectories by baseline BMI (time-by-baseline BMI *p* = 0.005; Table 3) was observed. Children with higher baseline BMI showed greater BMI increases over time. Model-adjusted estimates indicated that boys experienced an increase of 0.86 kg/m^2^ (95% CI: 0.51–1.21) during the intervention period (*p* < 0.001), which further increased to 1.64 kg/m^2^ (95% CI: 1.21–2.08) at the 3-year follow-up (*p* < 0.001). Girls increased during the intervention 0.92 kg/m^2^ (95% CI: 0.46–1.39; *p* = 0.845), while an increase of 1.54 kg/m^2^ (95% CI: 0.96–2.12; *p* < 0.001) was observed over the entire follow-up period.

Overall, during the post-intervention period (T3–T2) BMI increased by 0.70 kg/m^2^ (95% CI: 0.41–0.99; *p* < 0.001). No sex-by-time interaction was observed, indicating comparable changes in boys and girls (*p* = 0.861).

SDS-BMI remained stable across the study periods (time effect: F = 0.03, *p* = 0.967), with no evidence of a sex-by-time interaction (F = 0.03, *p* = 0.972) or modification by baseline SDS-BMI (baseline SDS-BMI–by–time interaction: F = 0.99, *p* = 0.375).

Model-adjusted mean changes were below 0.03 SDS-BMI units (all *p* ≥ 0.704), consistent with growth proportional to age- and sex-specific references. Accordingly, BMI-SDS trajectories did not differ by sex (F = 0.00, *p* = 0.963), and no differences were observed in post-intervention changes between boys and girls (T3–T2 sex contrast: 0. 199 SDS-BMI; *p* = 0.991). Overall, during the post-intervention period (T3–T2) SDS-BMI changed by +0.013 (95% CI: –0.178–1.09; *p* = 0.826).

Waist-to-height ratio showed modest increases over time (F = 3.33, *p* = 0.041), significantly modified by baseline WHR (WHR-by-time interaction F = 3.27, *p* = 0.044), but no sex-by-time interaction (F = 1.08, *p* = 0.347). Boys and girls followed similar waist-to-height ratio trajectories (sex effect F = 2.37, *p* = 0.131), with no differences in post-intervention changes (T2–T3 sex contrast –0.001, *p* = 0.934). Overall, post-intervention model-adjusted models showed a non-significant decrease of 0.0034 (95% CI: –0.010–0.017, *p* = 0.613) in waist-to-height ratio.

## 4. Discussion

This one-year post-intervention follow-up of the EDDY school-based nutrition and physical activity program showed that SDS-BMI remained stable. Our results contribute to the evidence gap in pediatric obesity prevention regarding children’s weight trajectories after the cessation of structured school-based health interventions. While short-term anthropometric benefits, although modest, are well-documented [5], sustained effects beyond active support remain underreported and inconsistent, particularly in European primary school settings [8]. The present analysis from the EDDY study provides novel data from Vienna and Austria, where such long-term follow-up studies are scarce.

The major finding from our study is the stability of SDS-BMI over a three-year follow-up period, despite absolute BMI increases consistent with normal growth. The model-adjusted mean BMI increase of 0.70 kg/m^2^ (95% CI: 0.41–0.99; *p* < 0.001) over one year, during the post-intervention period when children received no structured lifestyle intervention, is comparable to the variability in BMI change reported in the large pediatric cohort Bogalusa Heart Study (SD = 0.9 kg/m^2^ over one year) [26]. This modest increase in absolute BMI, together with the absence of an upward drift in SDS-BMI, may suggest a favorable outcome in terms of weight status stabilization following the intervention. It also corresponds to longitudinal trends previously described in normal-weight children [27], supporting the interpretation that fluctuations of this magnitude reflect physiological rather than pathological adiposity gain [26]. Nonetheless, our findings should be interpreted with caution given the absence of a control group.

We acknowledge that interpreting BMI changes in growing children is challenging without concurrent assessments of body composition. Nevertheless, although SDS-BMI does not directly measure fat or lean tissue, it remains a practical, standardized indicator whose reduction has been shown to reflect changes in adiposity, the tissue closely linked to metabolic complications [28].

Follow-up studies have indicated that reductions of at least 0.6 SDS-BMI in children with excessive weight are associated with reductions in body fat [28] while reductions of ≥0.25 SDS-BMI correspond to favorable improvements in cardiometabolic risk factors [29]. Although such thresholds are more often applied in clinical contexts to guide treatment goals, they are also relevant in school-based prevention. In Austria, school medical doctors could use SDS-BMI trajectories to identify children at elevated risk, initiate timely follow-up, and, when necessary, refer families for individualized weight-management support. Regular examinations by these school health professionals play an essential role in obesity prevention through the early detection of excessive weight gain and the facilitation of preventive counseling.

Current recommendations in childhood obesity prevention emphasize maintaining healthy, age- and sex-adjusted growth trajectories rather than focusing solely on weight reduction [13], consistent with our observed SDS-BMI stability. Although health-related quality of life was not reassessed at follow-up, earlier analyses of the EDDY program reported improvements immediately after the intervention [9]. Even though no causal inferences can be drawn, such prior improvements in health-related quality of life and self-efficacy may have encouraged the sustained adoption of healthy behaviors beyond the formal program [12]. Previous research also supports the role of psychosocial factors, including well-being and self-efficacy, in maintaining healthy behaviors over time [12,30,31]. Future studies incorporating repeated behavioral and psychosocial assessments would be valuable to elucidate potential mechanisms driving the observed anthropometric stability. Additionally, factors such as parental involvement and increased nutrition knowledge, as reported previously [7] may have contributed to the long-term maintenance of healthy habits. As previously assessed by our group regarding maternal perceptions of child weight status [32] and supported by evidence of both parents’ tendency to under-estimate overweight, especially in boys, with paternal education as a key modifier [33], mothers and fathers play critical yet complementary roles in shaping children’s health behaviors.

Within the school context, continued engagement by teachers in promoting healthy lifestyles may also help sustain outcomes, though this was not assessed directly and is likely influenced by workload and school [34].

Changes in waist-to-height ratio, a marker of central adiposity, were modest overall but varied according to baseline waist-to-height ratio, as indicated by a significant time-by-baseline interaction. This suggests that children with different initial levels of central adiposity may have followed distinct, though small, trajectories. These differences should be interpreted cautiously given the observational nature of the follow-up period. Although earlier reports often show greater relative benefits among children with elevated initial risk [17,18], median baseline and 3 year-follow-up waist-to-height ratio values in this group remained within normal limits. Nonetheless, such indicators could be of interest for school health doctors when identifying children who may benefit from closer monitoring or targeted preventive counseling. While mixed-model analyses revealed similar waist-to-height ratio trajectories between girls and boys, the modest sex difference observed over the full study period may reflect more pronounced waist-to-height ratio declines in girls during pre-pubertal years, potentially due to sex-specific fat distribution patterns or growth differences [35]. These differences in weight trajectories in obesity prevention programs are often not assessed [8].

This study has several methodological strengths, including standardized anthropometric measurements conducted by trained personnel, longitudinal mixed-effects modeling that accounted for baseline characteristics, and a high follow-up participation rate [8]. Furthermore, implementation within a routine school environment enhances ecological validity and real-world applicability. On the other hand, several limitations should be acknowledged. The absence of a control group during the follow-up period limits causal attribution of observed results to the intervention itself. In particular, without a concurrent control group, the apparent stability in SDS-BMI could partly reflect natural growth trends typical of this age group rather than a sustained effect of the intervention. The modest sample size may have reduced statistical power to detect smaller effects, and attrition was selective: Non-included participants were older and more often affected by excessive weight at the end of the intervention, possibly leading to conservative estimates of post-intervention development. Additionally, the lack of follow-up data on body composition and health behaviors restricts interpretation to anthropometric outcomes. Nevertheless, while some school-based health promotion programs have been reported in Austria [36,37], to our knowledge, no other study has yet reported similarly long-term follow-up data from a school-based obesity prevention program.

## 5. Conclusions

One year after the cessation of the EDDY intervention, participating children maintained stable SDS-BMI and age-appropriate waist-to-height trajectories. These findings support the potential of school-based nutrition and physical activity programs to promote the lasting normalization of growth patterns into adolescence. However, given the absence of a concurrent control group, these results should be interpreted with caution, as natural growth patterns cannot be entirely ruled out as an alternative explanation. Further controlled studies with extended follow-up and integrated behavioral measures are warranted to confirm long-term effectiveness and understand factors contributing to the maintenance of healthy trajectories.

## Figures and Tables

**Table 1:** Baseline characteristics of intervention group participants.

	All (n = 36)	Girls (n = 13)	Boys (n = 23)	*p* ^Ɨ^
Age (years), median (IQR)	7.91 (7.58–8.35)	7.98 (7.53–8.35)	7.89 (7.58–8.35)	0.908
BMI (kg/m^2^), median (IQR)	16.15 (14.80–17.40)	16.70 (15.40–17.30)	15.60 (14.80–17.50)	0.510
SDS-BMI, median (IQR)	0.036 (−0.612–0.767)	0.436 (−0.224–0.700)	−0.057 (−0.646–0.834)	0.479
Weight status, N (%)				
Normal weight	30 (83.3)	12 (92.3)	18 (78.3)	0.085
Overweight	5 (13.9)	0 (0)	5 (21.7)	
Obesity	1 (2.8)	1 (7.7)	0 (0)	
Waist circ. (cm), median (IQR)	56.50 (55.00–61.50)	55.00 (54.00–63.00)	57.00 (55.00–61.00)	0.632
WHR ratio, median (IQR)	0.447 (0.423–0.465)	0.447 (0.421–0.462)	0.448 (0.425–0.475)	0.882
Fat mass index, median (IQR)	3.25 (2.75–4.39)	3.97 (3.23–4.95)	2.91 (2.61–4.02)	0.103
EW (hh:mm/day), median (IQR)	11:30 (11:00–12:10)	11:05 (11:00–11:30)	11:41 (11:00–12:30)	0.329
Breakfast intake, N (%)				
Regular	11 (30.6)	3 (23.1)	8 (34.8)	0.708
Irregular	25 (69.4)	10 (76.9)	15 (65.2)	
Parental background, N (%)				
Both parents from Austria	8 (22.2)	6 (26.1)	2 (15.4)	0.851
One parent from Austria	3 (8.3)	2 (8.7)	1 (7.7)	
No parent from Austria	25 (69.4)	15 (65.2)	10 (76.9)	
Mother’s BMI, (kg/m^2^) ^1^	24.68 (22.66–28.13)	24.65 (22.13–29.38)	24.74 (23.34–26.67)	0.918
Father’s BMI, (kg/m^2^) ^1^	25.95 (24.31–27.77)	26.47 (24.76–29.56)	25.93 (22.89–26.42)	0.410
Maternal nutritional status, N (%)				
Non-excessive weight ^2^	19 (54.3)	7 (53.9)	12 (54.6)	0.968
Excessive weight ^2^	16 (45.7)	6 (46.2)	10 (45.5)	

Values indicate N (%) for categorical variables and median (interquartile range, IQR) for continuous variables. References: IQR: interquartile range; BMI: body mass index; WHR: waist-to-height ratio; EW: eating window. ^Ɨ^ Chi-square (or Fisher) tests were used for categorical variables and Mann–Whitney U test to compare the distributions of continuous variables across sex categories. ^1^ Based on reported data from n = 35 mothers and n = 33 fathers. ^2^ Non-excessive weight comprises low and normal weight categories, while excessive weight includes overweight/obesity according to BMI classification.

**Table 2:** Median (IQR) anthropometric changes by sex across study periods.

Outcome	Period	Girls	Boys	*p* *	Z	*r*_rb_
BMI (kg/m^2^)	T2–T1 (intervention)	0.70 (0.00–2.10)	0.50 (0.10–1.70)	0.717	0.347	0.058
	T3–T2 (post-intervention)	0.60 (0.00–1.20)	0.50 (0.20–1.50)	0.586	−0.523	−0.088
	T3–T1 (3y follow-up)	1.60 (0.70–2.00)	0.90 (0.60–2.20)	0.729	0.330	0.055
SDS-BMI	T2–T1 (intervention)	−0.181 (−0.361–0.226)	−0.102 (−0.276–0.230)	0.856	−0.165	−0.027
	T3–T2 (post-intervention)	−0.001 (−0.114–0.106)	0.022 (−0.216–0.225)	0.856	−0.165	−0.027
	T3–T1 (3y follow-up)	−0.069 (−0.247–0.304)	−0.174 (–0.331–0.240)	0.780	0.264	0.044
WHR	T2–T1 (intervention)	0.015 (−0.037–0.034)	0.008 (−0.008–0.032)	0.564	−0.560	−0.093
	T3–T2 (post-intervention)	0.009 (−0.027–0.030)	0.008 (−0.007–0.017)	0.882	−0.132	−0.022
	T3–T1 (3y follow-up)	−0.023 (−0.045–0.000)	0.010 (−0.011–0.036)	0.050	−1.943	−0.324

References: BMI: Body mass index; WHR: Waist-to-height ratio. Changes are expressed as median (interquartile range, IQR) absolute changes between baseline and the end of the intervention (T2–T1), between the end of the intervention and 1-year follow-up (T3–T2), and across the full study period (T3–T1). * *p*-values refer to differences between girls and boys assessed using the Mann–Whitney U test. Z denotes the standardized Wilcoxon rank-sum statistic, and rank-biserial correlations (*r*_rb_ = Z/√N) were used as effect size measures for sex differences.

**Table 3:** Mixed models results for anthropometric changes.

	F-Value, (*p*-Value)			
	Time	Sex	Time-by-Sex Interaction	Time-by-Baseline Outcome Interaction
BMI	2.26 (0.112)	0.00 (0.964)	0.15 (0.861)	5.88 (0.005)
BMI-SDS	0.03 (0.967)	0.00 (0.963)	0.03 (0.972)	0.99 (0.375)
WHR	3.33 (0.041)	2.37 (0.131)	1.08 (0.347)	3.27 (0.044)

References: BMI: Body mass index; WHR: Waist-to-height ratio. Linear mixed-effects models for repeated measures were employed. The factors time (baseline, end of the intervention and follow-up after the intervention ended), sex (girls and boys), and baseline age and anthropometric outcomes (BMI, SDS-BMI, and waist-to-height ratio) and their interaction were treated as fixed effects. Participants were treated as random effects to account for potential clustering.

## Data Availability

The data presented in this study are available on request from the corresponding author due to ethical reasons.

## Supplementary Materials

The following supporting information can be downloaded at: https://www.mdpi.com/article/10.3390/children13040485/s1; Table S1: Anthropometric characteristics at intervention end (T2) for included and excluded participants at one-year post-intervention follow-up.

## Abbreviations

The following abbreviations are used in this manuscript:

EDDY	Effect of sports and diet training to prevent obesity and secondary diseases and to influence young children’s lifestyle
BMI	Body mass index
WHR	Waist-to-height ratio
IQR	Interquartile range
EW	Eating window
