# Supplementary material for: One-Year Follow-Up of Weight Trajectories After the EDDY School-Based Nutrition and Physical Activity Program
Source: Children (Basel). 2026 Mar 31;13(4):485. doi: 10.3390/children13040485 (PMC13115548; doi:10.3390/children13040485)
Supplement: Supplementary file 1 [file children-13-00485-s001.zip › children-4197322-supplementary.pdf]

Supplementary Material

One-Year Follow-up of Weight Trajectories after the EDDY School-Based Nutrition and Physical Activity Program

Table S1. Anthropometric characteristics at intervention end (T2) for included and excluded participants at one-year post-intervention follow-up.

	**Included (n=36)**	**Excluded (n=12)**	p^{*}	**Excluded girls (n=4)**	**Excluded boys (n=8)**	$p^{ɫ}$
Age (years), median (IQR)	9.56 (9.23-9.99)	9.98 (9.83-10.49)	0.004	9.98 (9.89-10.23)	9.94 (9.80-10.71)	0.999
BMI (kg/m^2), median (IQR)	16.85 (15.30-19.05)	17.25 (16.00-21.55)	0.253	18.00 (16.20-21.10)	16.60 (16.00-21.85)	0.999
SDS-BMI, median (IQR)	−0.030 (−0.60-0.92)	0.20 (−0.45-1.47)	0.366	0.46 (-0.32-1.34)	−0.086 (−0.45-1.48)	0.999
Weight status, N (%)						
Normal weight	28 (77.8)	9 (75.0)	0.030	3 (75.0)	6 (75.0)	0.999
Overweight	7 (19.4)	0 (0)		0 (0)	0 (0)	
Obesity	1 (2.8)	3 (25.0)		1 (25.0)	2 (25.0)	
WHR, median (IQR)	0.45 (0.41-0.49)	0.45 (0.42-0.52)	0.609	0.44 (0.41-0.49)	0.45 (0.43-0.55)	0.497
Fat mass index, median (IQR)	3.36 (2.48-4.38)	3.66 (2.89-6.90)	0.253	4.19 (3.45-6.52)	3.27 (2.65-7.04)	0.610

References: BMI: body mass index; WHR: waist-to-height ratio. Variables correspond to measurements at the end of the intervention period, after 2 years of follow-up (T2). * p for the difference between included (n=36) and excluded (n=12) participants, using Mann-Whitney U test for continous variables and Chi-square for categorical variables. ^{ɫ} p for the difference between sex among the excluded participants (n=12), using Mann-Whitney U test for continous variables and Chi-square for categorical variables.
